# The effects of cognitive behavioral therapy for insomnia in people with type 2 diabetes mellitus, pilot RCT part II: diabetes health outcomes

**DOI:** 10.1186/s12902-020-00612-6

**Published:** 2020-09-05

**Authors:** Mohammed M. Alshehri, Shaima A. Alothman, Aqeel M. Alenazi, Jason L. Rucker, Milind A. Phadnis, John M. Miles, Catherine F. Siengsukon, Patricia M. Kluding

**Affiliations:** 1 Physical Therapy and Rehabilitation Science Department, University of Kansas Medical Center, 3901 Rainbow Blvd., Mail Stop 2002, Kansas City, Kansas 66160 USA; 2 Physical Therapy Department, Jazan University, Jazan, Southern Region Saudi Arabia; 3 Lifestyle and Health Research Center, Princess Nora bint Abdulrahman University, Riyadh, Saudi Arabia; 4 Physical Therapy Department, Prince Sattam Bin Abdulaziz University, Alkharj, Central Region Saudi Arabia; 5 Department of Biostatistics, University of Kansas Medical Center, Kansas City, Kansas USA; 6 Endocrinology Department, University of Kansas Medical Center, Kansas City, Kansas USA

**Keywords:** Insomnia, Diabetes, Cognitive behavioral therapy, Self-care, Glycemic control

## Abstract

**Background:**

Previous studies have shown the negative impact of sleep disturbances, specifically insomnia symptoms, on glucose metabolism for people with type 2 diabetes (T2D). People with insomnia symptoms are at risk of poor glycemic control and suboptimal diabetes self-care behavior (DSCB). Investigating the impact of a safe and effective intervention for individuals with T2D and insomnia symptoms on diabetes’ health outcomes is needed. Therefore, the aim of this exploratory study is to examine the effects of Cognitive Behavioral Therapy for Insomnia (CBT-I) on glycemic control, DSCB, and fatigue.

**Methods:**

Twenty-eight participants with T2D and insomnia symptoms, after passing an eligibility criteria at a medical research center, were randomly assigned to CBT-I (*n* = 14) or Health Education (HE; *n* = 14). The CBT-I and HE groups received 6 weekly one-hour sessions. This Randomized Controlled Trial (RCT) used a non-inferiority framework to test the effectiveness of CBT-I. Validated assessments were administered at baseline and post-intervention to assess glycemic control, DSCB, and fatigue. A Wilcoxon signed-rank test was utilized to compare within-group changes from baseline to post-intervention. A Mann-Whitney test was utilized to measure the between-group differences. Linear regression was used to assess the association between the blood glucose level and the number of days in the CBT-I group.

**Results:**

The recruitment duration was from October 2018 to May 2019. A total of 13 participants completed the interventions in each group and are included in the final analysis. No adverse events, because of being a part of this RCT, were reported. CBT-I participants showed significantly greater improvement in glycemic control, DSCB, and fatigue. There was a significant association between the number of days in the CBT-I intervention with the blood glucose level before bedtime (B = -0.56, *p* = .009) and after awakening in the morning (B = -0.57, *p* = .007).

**Conclusions:**

This study demonstrated a clinically meaningful effect of CBT-I on glycemic control in people with T2D and insomnia symptoms. Also, CBT-I positively impacted daytime functioning, including DSCB and fatigue. Future research is needed to investigate the long-term effects of CBT-I on laboratory tests of glycemic control and to understand the underlying mechanisms of any improvements.

**Trial registration:**

Clinical Trials Registry (NCT03713996). Retrospectively registered on 22 October 2018,

## Background

A systematic review and meta-analysis of epidemiological studies found that people with type 2 diabetes (T2D) and sleep disturbances were more likely at risk of poor glycemic control as measured by glycated hemoglobin (HbA1c) and suboptimal diabetes self-care behavior (DSCB) [1, 2]. A recent study found that people with T2D and insomnia symptoms had worse scores in several health domains related to DSCB compared to people with T2D without insomnia symptoms [3]. Additionally, increases in insomnia severity was associated with suboptimal DSCB among people with T2D [3]. It is possible that sleep disturbances lead to fatigue and physical inactivity, which then contributes to poor diabetes health outcomes [4].

Previous studies have shown that insomnia symptoms are common among people with T2D [5, 6], and insomnia itself may independently increase mortality rates [7, 8]. In addition, the increased mortality risk in people with insomnia might be due to inflammation, which is also associated with cardiovascular diseases [9]. The mechanisms underlying the relationship between T2D and insomnia symptoms are not well understood [10, 11], yet there is a need to identify an effective treatment for insomnia symptoms to improve T2D health outcomes.

Among therapeutic options for insomnia, the American Academy of Sleep Medicine recommends the employment of Cognitive Behavioral Therapy for Insomnia (CBT-I) as first-line therapy [12], since CBT-I can be superior when compared to sleep medications [13]. These sleep medications can possibly lead to negative side effects [14] or metabolic alterations [15, 16]. Further, a recent study supported the need to investigate the effects of CBT-I on people with T2D due to the harmful side effects of pharmacological treatments and the limited evidence of effectiveness [17].

CBT-I is a potentially efficacious intervention for people with T2D as it may address an altered metabolism. Generally, the components of CBT-I including sleep restriction therapy, stimulus control therapy, cognitive therapy, and relaxation therapy strengthen the association between bed and sleep connection, readjust the homeostatic mechanisms and the circadian rhythm, and reduce anxiety and rumination about sleep [18]. CBT-I modules may interrupt the physiological mechanisms such as hypothalamic pituitary-adrenal (HPA) axis activation [10, 11], which may be one link between insomnia symptoms and T2D. For example, it has been shown that an association between sleep homeostasis and glucose regulation could be adjusted using sleep restrictions and stimulus control therapies [19]. Improvement of the circadian cycle misalignment has intimate correlations with glucose metabolism for people with T2D [20]; however, this has not been proven yet in CBT-I studies. In addition, ancillary modules in CBT-I, such as relaxation techniques and sleep hygiene, could play a role in reducing stress and nocturia episodes (i.e., the number of bathroom visits per night) [21–23].

The objective of this study was to explore the effects of 6 sessions of CBT-I on HbA1c, DSCB, and fatigue. We hypothesized that the participants in the CBT-I group would have greater improvements in HbA1c, DSCB, and fatigue compared to the participants in the health education (HE) group. The effects of CBT-I on sleep outcomes were reported separately (Part I of the intervention trial: Using Cognitive Behavioral Therapy for Insomnia in People with T2D, Pilot RCT Part I: Sleep Outcomes and Concomitant Symptoms) [24]. We anticipated improvements in sleep and concomitant outcomes which will positively impact people with T2D and health outcomes because of the relationship between insomnia symptoms and poor diabetes-related health outcomes. Both intervention studies were designed to serve as pilots for a subsequent larger scale intervention trial.

## Methods

### Participants and materials

The procedures and interventions for this project were described in a published protocol report [25]. This intervention trial was described according to the CONSORT 2010 guidelines [26]. Prior to being enrolled in the study, potential participants were screened according to eligibility criteria, which are presented in Table 1.

**Table 1:** The inclusion and exclusion criteria

I**nclusion Criteria**	**Exclusion Criteria**
Ages between 40 to 75 years	Self-reported neurological diseases (e.g. Alzheimer’s disease, Parkinson’s disease, Traumatic Brain Injury, Stroke, Multiple Sclerosis)
Self-reported diagnosis of type 2 diabetes	Self-reported untreated sleep disorders as well as: - Scored > 4 on Stop-Bang score - Failed to pass Restless Leg Syndrome Diagnostic Index
Scored > 10 on Insomnia Severity Index and self-reported symptoms of insomnia at least 3 nights/week for the past 3 months	Scored ≥7 on Brief Pain Inventory
Able to travel to the University of Kansas Medical Center to attend 6 sessions	Scored ≥21 on Beck Depression Scale
Able to understand and follow verbal commands in English	Scored ≥15 on Generalized Anxiety Disorder-7
	Self-reported following medical issues: Chronic Fatigue Syndrome, Fibromyalgia, Bipolar, Seizure Disorders and Rheumatic Diseases, Dialysis, blindness, trans-femoral amputation, speech deficits, or significant auditory impairment
	Performed night shift work
	Heavy alcohol drinker (≥15 alcohol drinks per week for men and ≥ 8 alcohol drinks per week for women)
	Reported being pregnant

### Study design

This RCT had an allocation ratio of 1:1 and utilized a superiority framework to test the effectiveness of the CBT-I. Participants were randomly assigned to either the CBT-I group (*n* = 14) or the HE group (*n* = 14). We used age to stratify participants into either the older (63–75 years) or the younger (40–62 years) age group. This study was retrospectively registered in the Clinical Trials Registry (NCT03713996) [27]. This study was approved by the Institutional Review Board and the Human Subjects Committee of the University of Kansas Medical Center. All participants signed a written informed consent before the assessment visit. Data collections and provided interventions took place at the University of Kansas Medical Center.

### Outcomes

All participants completed outcome measures at the baseline, and all participants completed the same outcome measures 1 week after completing the intervention. The primary outcome, insomnia severity, was included in the RCT Part I in which the power calculation was established and its preliminary data were published elsewhere [24].

#### Diabetes control measurement

A point-of-care instrument was used to assess HbA1c using a disposable finger stick HbA1c kit (A1CNow + test kit; Bayer Healthcare, Tarrytown, NY). This instrument measures the level of glycosylated hemoglobin via an immunoassay test, and reflects the average glucose blood levels over the period of 6 to 12 weeks [28]. During a previous diabetes management program, the A1CNow + provided accuracy and precision when performing a point-of-care, and a 0.05 reduction in HbA1c is considered clinically meaningful [29]. In addition, random blood glucose (RBG) levels were assessed by a glucose meter (FreeStyle Flash, Contour® Bayer Healthcare, Diagnostic Division, Tarrytown, NY). Participants were tested for the RBG without time specifications or diet instructions. During the intervention, participants in the CBT-I group were asked to record their own blood glucose levels right before bedtime and after first awakening in the morning throughout the study period (i.e., 7 days/nights per week for 7 weeks).

#### Diabetes self-care behavior (DSCB)

Self-care was assessed using the Diabetes Care Profile (DCP), which is a validated survey that measures 13 psychosocial and educational factors [30, 31]. The 13 domains that are associated with the management of diabetes, are inclusive of the following: understanding the management of practice, support, control problems, social and personal factors, positive attitude, negative attitude, care ability, importance of care, self-care adherence, diet adherence, long-term care benefits, exercise barriers, and glucose monitoring barriers [31]. A standardized total DCP composite score was established to present all 13 domains that were scored according to the Fitzgerald et al. scoring criteria [31]. Next, each participant’s domain score was standardized using z-scores, and then averaged to create a standardized total DCP composite score. High scores on the DCP composite score indicate better DSCB.

#### Fatigue severity

Daily fatigue was measured using the Fatigue Severity Scale (FSS) that consists of 9 items developed to assess disabling fatigue on daily life. The FSS has been shown to be valid and reliable [32]. Each item was measured on a 7-point Likert scale ranging from 1 (strongly disagree) to 7 (strongly agree). Mean item response for the completed FSS items was used for analysis.

### Interventions

All participants in the CBT-I group and HE group attended 6 sessions that were scheduled consistently one session per week with the CBT-I provider. These sessions were provided for around 45 min for both groups to assure all participants received the same amount of attention. Neither the CBT-I provider nor the participants were blinded in this study. The protocol paper describes session by session of both interventions [25].

#### Cognitive behavioral therapy for insomnia

This protocol intervention was designed based on a session-by-session guide [33]. Five main therapeutic techniques were provided during the 6-sessions including sleep restriction therapy, stimulus control therapy, sleep hygiene, relaxation techniques, and cognitive therapy. In order to monitor nightly sleep changes and issues, the CBT-I provider reviewed the sleep diary for each session. In addition, calculations in sleep changes were made to prescribe the sleep schedules for the following week. The calculations in the sleep changes were made based on the sleep efficiency (the ratio of total sleep time and total bedtime multiplied by 100) from the weekly sleep diary. At each session, the time spent in bed and out of bed was prescribed based on the calculation of sleep efficiency in percentages. If the sleep efficiency was greater than 90%, the opportunity to go to bed earlier was extended by 15 min. If the sleep efficiency was between 85 and 89.9%, the same sleep schedule was prescribed, and if it was less than 85%, then bedtime moved 15 min later.

Session 1: Sleep restriction therapy, stimulus control therapy and sleep hygiene were provided. Sleep restriction therapy aligns the time in bed with the total sleep time by identifying the wake time and total sleep time needed to increase the sleep efficiency [34, 35]. Stimulus control associates the bed environment to sleep only (or sex) to reinforce the circadian rhythm [34, 35]. In addition, sleep hygiene minimizes the influence of negative behaviors on sleep quality and quantity. The principles of sleep hygiene were provided including the impact of diet, exercise, caffeine, alcohol, and environment on the quality of sleep [34, 35].

Session 2: Calculating sleep efficiency, reviewing the principles of sleep hygiene, and providing the diaphragmatic breathing technique were covered in this session. During this week, we also reviewed the sleep diary to confirm any necessary sleep changes (i.e., adjustments to the time both in and out of bed.) The diaphragmatic breathing technique promotes muscle relaxation, better breathing performance, and memory relaxation [36]. It also was especially emphasized for those who were not able to relax.

Session 3: Calculating both sleep efficiency and mindfulness were conducted during this session. Mindfulness reduces cognitive and somatic arousal for people with insomnia who received CBT-I [37]. The principles of mindfulness including how to be non-judgmental and incorporate patience, trust, acceptance, and letting go were covered in this session.

Session 4: Calculating sleep efficiency and progressive muscle relaxation were reviewed in this session. Muscle relaxation therapy helped in improving insomnia and depression symptoms when used in CBT-I [38]. Muscle relaxation therapy is a physiological intervention aimed to assess and decrease muscle tension [38].

Session 5: Calculating sleep efficiency and cognitive therapy were done in this session. Cognitive therapy changes both detrimental beliefs and attitudes about sleep. During this session, we worked on reducing the participants’ sleep efforts, catastrophizing, and their anxiety about sleep, while also working on their willingness to modify their sleep-related behaviors and engage in good sleep strategies.

Session 6: Assessing treatment benefits and insomnia relapse education were provided in this session. We graphically reviewed the sleep efficiency of each session to show the sleep changes during the six sessions of CBT-I. In addition, we discussed the approaches to maintain clinical gains and how to remedy any incidents when insomnia returns.

#### Health education

Five main health education materials were introduced during the six sessions including brief sleep hygiene, foot care, diabetes classifications, healthy diet, and physical activity. During the HE sessions, we provided informal face to face interviews to better engage the participants in the conversations. Participants’ comprehension of and experiences about the provided materials were facilitated through open questions. Details of the provided sessions for the HE group were provided in the protocol paper of this study [25].

### Statistical analysis

All data analyses were performed using SPSS 23.0 for Mac (Chicago, IL) and R (https://www.R-project.org/) [39]. Descriptive statistics included means and standard deviations for the assessed variables at the baseline (14 participants) and at the post-assessment (13 participants). We used Shapiro–Wilk tests to assess the normality of residuals during model development. For the main analysis, we used Mann-Whitney U tests to examine the between-group differences of the CBT-I and HE groups in HbA1c, RBG, DSCB, and fatigue mean change scores for those who completed the study. We also used Wilcoxon signed-rank tests to compare the within-group changes for both groups. Effect sizes were calculated using Cohen’s *d* [40]. For graphical purposes, we calculated absolute percentage changes in all outcomes to graph the between-group differences. For secondary purpose, we used linear regression analyses to predict blood glucose levels (before bedtime and after first awakening in the morning) based on 49 days throughout the course of the study, including 6 weeks CBT-I and post-assessment. For all analyses, the alpha level was set at .05.

## Results

**Fig. 1:**
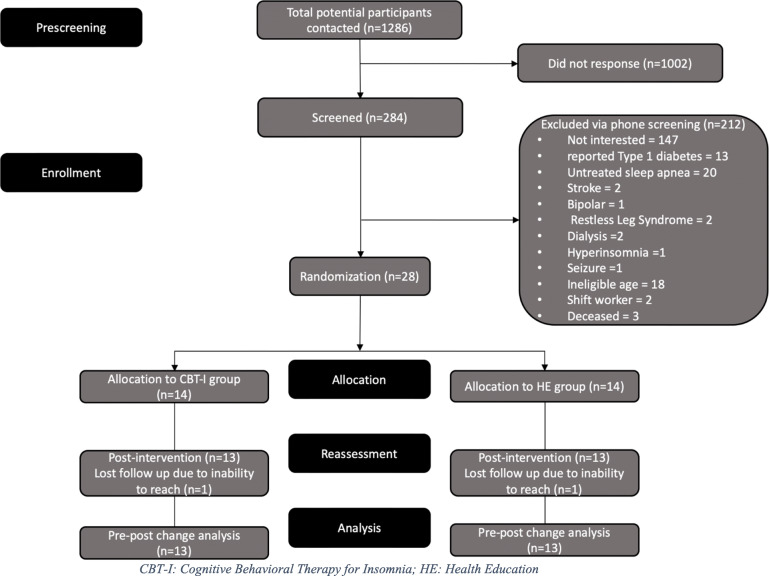
Consort of the study

The consort of this intervention trial shows a total of 28 participants enrolled in the study and 26 participants completed the study (Fig. 1). There were no baseline differences between groups in demographics including age, sex, ethnicity, and education (*p* > .05) [24]. In addition, there were no significant between-group differences in the baseline assessments of HbA1c, RBG, DCP composite score, or FSS (Table 2).

**Table 2:** Comparison of clinical variables within and between groups

	**CBT-I (mean ± SD)**		***p***^***a***^	**HE (mean ± SD)**		***p***^***a***^	***p***^***b***^	***p***^***c***^
	**Pre, *n*** **= 14**	**Post, *n*** **= 13**		**Pre, *n*** **= 14**	**Post, *n* = 13**			
**HbA1c, %**	7.8 ± 2.1	7.3 ± 1.8	.02	6.5 ± 0.6	6.7 ± 0.8	.19	.09	.01
**RBG**	177.46 ± 110.97	154.70 ± 38.72	.91	137.00 ± 19.16	144.46 ± 30.68	.43	.66	.58
**DCP**	−0.21 ± 0.53	0.19 ± 0.40	.03	− 0.32 ± 0.44	− 0.28 ± 0.52	.65	.80	.01
**FSS**	4.20 ± 1.40	2.79 ± 1.21	.002	4.36 ± 1.44	4.30 ± 1.58	.56	.95	.001

*CBT-I* Cognitive Behavioral Therapy for Insomnia, *HE* Health Education, *A1C* Glycemic control, *RBG* Random blood glucose, *DCP* Diabetes Care Profile composite score, *FSS* Fatigue Severity Scale; ^a^Comparison of the pre- and post-intervention values using ^a^Wilcoxon signed-rank test; ^b^Baseline difference between groups; ^c^Comparison of between group difference using Mann-Whitney U tests

**Fig. 2:**
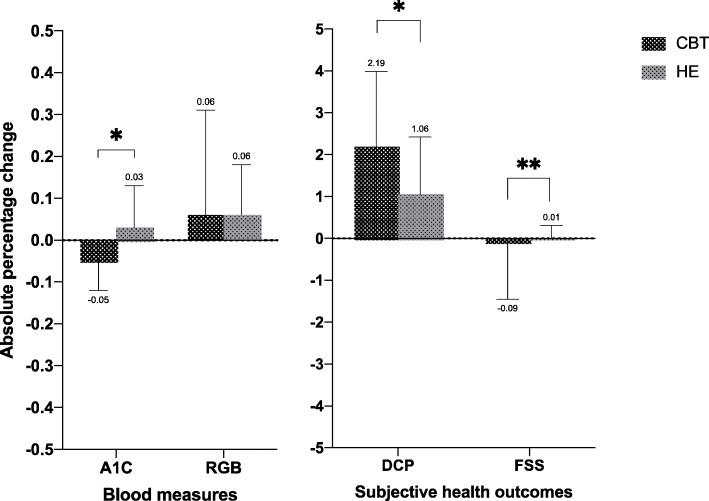
Absolute percentage change of all outcomes for both groups; *p = 0.01, **p = 0.001

There were significant between-group post-intervention differences in HbA1c (*d* = 0.41, *p* = .01), DCP composite score (*d* = 1.01, *p* = .01), and FSS (*d* = 1.07, *p* = .009; Fig. 2; Table 2). There were significant within-group differences for the CBT-I group in HbA1c (*p* = .02), DCP composite score (*p* = .03), and FSS (*p* = .002), which are also shown in Table 2. However, there were no within-group differences in HbA1c, DCP composite score, or FSS for the HE group.

**Fig. 3:**
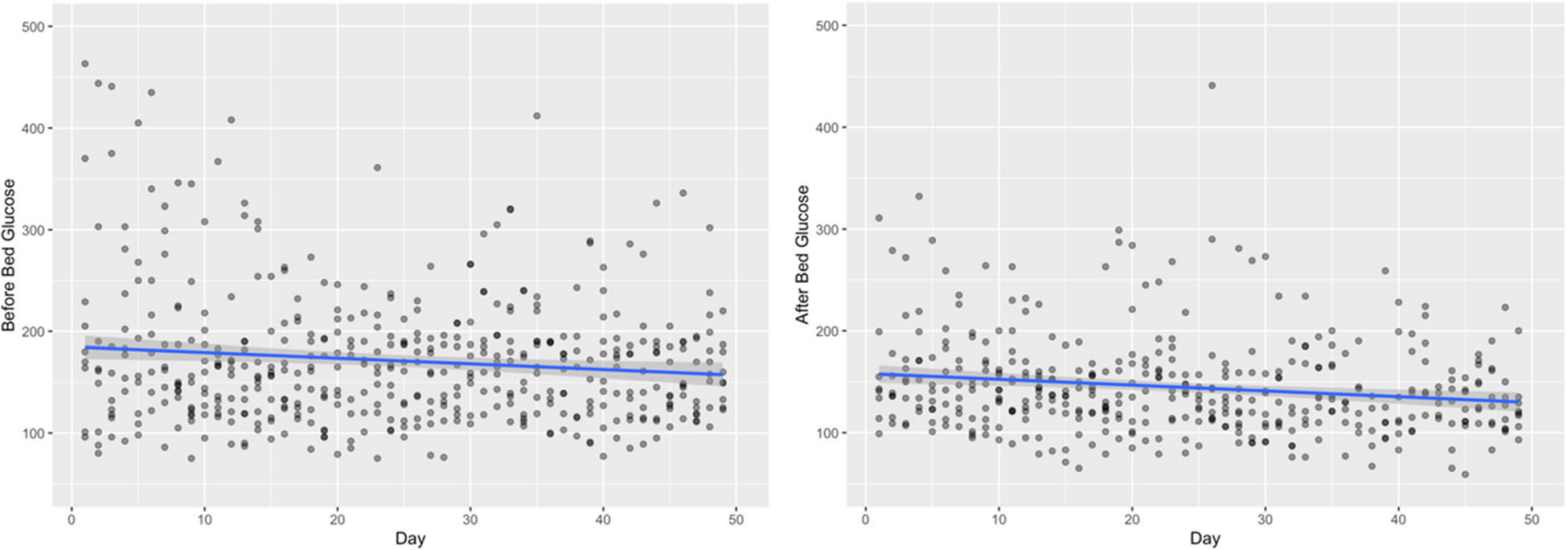
Daily glucose blood levels before bedtime and after awakening in the morning during the CBT-I intervention

We noted declines in the blood glucose levels before bedtime and after first awakening in the morning for the CBT-I group using the R software package (Fig. 3). The linear regression analysis showed significant association between the number of days in the CBT-I intervention with a blood glucose level before bedtime (B = -0.56, *p* = .009) and after first awakening in the morning (B = -0.57, *p* = .007) (Fig. 3).

## Discussion

To the best of our knowledge, this was the first RCT to examine the effects of CBT-I on diabetes outcomes and daytime functioning in people with T2D and insomnia symptoms. This study suggested CBT-I was effective in improving HbA1c, DSCB, and FSS for people with T2D and insomnia symptoms. Glucose blood levels, both before bedtime and after first awakening in the morning, also decreased over the course of the CBT-I intervention.

Diabetes outcomes improved following CBT-I, with a clinically meaningful difference in HbA1c. Clinical improvement in HbA1c may have been due to reductions in insomnia severity or psychological symptoms, which might foster an improved DSCB. After the CBT-I intervention, as shown in Fig. 2, there was a 0.05% absolute percentage reduction in HbA1c that suggests a clinically significant change based on the American Diabetes Association statistics [41]. It is recommended that people with T2D maintain HbA1c levels below 6% to reduce their risk of developing microvascular complications, although HbA1c between 6.5 and 7.9% is often considered acceptable by physicians [42]. Interestingly, the HE intervention provided to the control group, which included sleep hygiene, diet, and physical activity, was not sufficient to improve HbA1c. However, the baseline data of HbA1c for people in the HE group were at the borderline of optimum glycemic control that has been recommended by the American Diabetes Association. Thus, future research is needed to consider HbA1c in the power calculation and randomization process. As shown in the initial part of this intervention trial, improving insomnia symptoms following CBT-I may produce reductions in depression and anxiety symptoms that are often associated with daily hyperglycemia [24]. Previous studies have shown the negative influence of the combination of insomnia and depression on an individual’s glucose metabolism [43], which could be adjusted using CBT-I [44].

Besides the effects of improving insomnia symptoms on HbA1c, improvements in DSCB could also explain the clinical changes in HbA1c. Our study tracked glucose levels for participants in the CBT-I group, and there was a statistically significant decrease over the course of the intervention. This is entirely observational, however, as we did not monitor glucose before bedtime and after first awakening in the morning in the HE group. Regardless, self-monitoring of blood glucose should be done as a part of DSCB when trying to minimize problems related to hyperglycemia. It has been suggested that self-monitoring of blood glucose significantly reduces HbA1c levels for people with poorly controlled T2D [45].

There are few physiologic mechanisms that might explain improvements in HbA1c. First, the negative effects of sleep disturbances on metabolism might cause decreased brain glucose utilization, which could lead to hyperglycemia [46]. Reducing sleep disturbances via CBT-I might regulate glucose utilization, which could improve HbA1c. Second, previous studies have suggested a U-shaped relationship between sleep duration with HbA1c levels, where excessively short or long sleep durations have been noted to be associated with higher HbA1c levels. Sleep restriction therapy might lead to improved HbA1c levels by maintaining sleep durations within an optimal range of 7–8 h. Third, sleep disturbances are associated with appetite hormone dysregulations [46], and these dysregulations could be adjusted through sleep hygiene and stimulus control therapy. Sleep hygiene and stimulus control might help the participants in scheduling meals and acquiring a better understanding of their bodily needs regarding food consumption. Fourth, abnormal HPA axis activation might be normalized as a result of improving insomnia symptoms. This normalization could reduce cortisol secretion during sleep, which has been linked to reduced morning glucose levels. Finally, people with poor sleep and T2D at risk of impaired decision-making [47], which might be improved following CBT-I. Effective decision making may assist people with T2D in understanding domains related to diabetes such as food choices, control problems, diabetes distress, and medication adherence.

A population-based RCT of 109 older adults with insomnia showed the risk of 8 biomarkers of various health conditions following mood and daytime functioning promotions as a part of cognitive behavioral therapy [48]. The authors suggested that improving sleep quality was associated with reducing the risk of cardiovascular, metabolic, and inflammatory diseases [48]. As mentioned previously, the possible changes in the physiological systems, might be associated with an increased allostatic load, which was related to diabetes, hypertension, metabolic syndrome, and cardiovascular disease [48]. This suggests sleep promotion programs may help in re-establishing healthy patterns of various biomarkers for various health conditions due to the association between the circadian clock regulation and the rhythms of body organs or hormonal secretory patterns that alter numerous biological processes such as metabolism and blood glucose regulation [46]. The current study adds to the literature in regard to the benefits of CBT-I on an important indicator of diabetes control, HbA1c, in people with T2D. Part I of this intervention trial approved the effects of CBT-I on various sleep parameters and psychological symptoms which might indirectly show a positive impact on diabetes health outcomes. However, future study is needed to investigate the effect of CBT-I on a wide range of metabolism parameters for people with T2D.

Although the HE group received the same amount of face-to-face attention, we did not find any significant improvement in their diabetes and daytime functioning outcomes. This reiterated the importance of considering CBT-I as a treatment in diabetes clinics for people with T2D who suffer from insomnia symptoms. Contrary to the positive results of CBT-I on insomnia symptoms, glycemic control, and fatigue in people with T2D, there were no significant improvements in RBG for either group. Several factors might explain these results such as the specificity of interventions, the short-term intervention, or the methodological factors. CBT-I is designed to change detrimental beliefs about sleep behavior, which could demonstrate a secondary effect on RBG over time. However, the data from this study suggests blood glucose levels could be decreased at night before bedtime and in the mornings after first awakening.

This study has identified the effects of CBT-I on diabetes health outcomes in people with T2D; however, some limitations need to be considered for future research. First, including other highly sensitive glucose metabolism measures on larger sample sizes, such as a homeostatic model assessment and an oral glucose tolerance test, may generalize the other effects of CBT-I on diabetes parameters. Second, diabetes management includes interdisciplinary approaches, such as diet, physical activity, and medication adherence, to ensure optimal HbA1c. Future work needs to track daily changes in these activities to better explain the impact of CBT-I on HbA1c. Third, comprehensive functional assessments, including variables such as cognition, motivation, and activities of daily living, may help to efficiently identify other results following CBT-I. Fourth, we were not able to determine if the improvement in HbA1c was mediated by a reduced severity of insomnia or psychological symptoms. A longitudinal design for a larger sample size with T2D is needed to better understand the factors that promote good glycemic control following CBT-I. Finally, although this study demonstrated clinical improvements in HbA1c and DSCB after participants underwent six sessions of CBT-I, future research is needed to measure the sustainability of these improvements for at least 3 months.

## Conclusion

This study focuses on clinical information about the effectiveness of CBT-I on diabetes health outcomes. CBT-I showed a clinically meaningful effect on HbA1c and significant improvements in optimal DSCB and fatigue in people with T2D and insomnia symptoms. There is still a need to understand the underlying mechanism of these enhancements, and future research is needed to investigate the long-term effect of CBT-I on diabetes blood parameters and to understand the underlying mechanisms of these improvements.

## Supplementary information

**Additional file 1.**

## Data Availability

The datasets analyzed during the current study are available from the corresponding author upon reasonable request.

## Abbreviations

RCT: Randomized Controlled Trial
T2D: Type 2 Diabetes
HPA: Hypothalamic Pituitary-adrenal
CBT-I: Cognitive Behavioral Therapy for Insomnia
HE: Health Education
HbA1c: Glycemic Control
RBG: Random Blood Glucose
DSCB: Diabetes Self-care Behavior
DCP: Diabetes Care Profile
FSS: Fatigue Severity Scale
